# Late onset schizophrenia and delusional disorder: activity of platelet energy, glutamate, and glutathione metabolizing enzymes

**DOI:** 10.1192/j.eurpsy.2022.2011

**Published:** 2022-09-01

**Authors:** V. Pochueva, O. Savushkina, I. Boksha, E. Tereshkina, T. Prokhorova, V. Sheshenin, E. Vorobyeva, G. Burbaeva

**Affiliations:** 1 FSBSI “Mental Health Research Centre”, Geriatric Psychiatry, Moscow, Russian Federation; 2 FSBSI “Mental Health Research Centre”, Laboratory Of Neurochemistry, Moscow, Russian Federation

**Keywords:** glutamate dehydrogenase, glutathione reductase, glutathione-S- transferase, late onset schizophrenia

## Abstract

**Introduction:**

Alterations of glutamate, energy and glutathione metabolism contribute to the pathogenesis of psychotic disorders

**Objectives:**

Revealing clinical-biological correlations in patients with late onset schizophrenia and delusional disorder by determining clinical parameters and activity of platelet enzymes of energy, glutamate, and glutathione metabolism.

**Methods:**

27 women of 45-80 years old were studied, with late onset schizophrenia or delusional disorder. Activity of platelet cytochrome *c*-oxidase (COX), glutamate dehydrogenase (GDH), glutathione reductase (GR) and glutathione-S-transferase (GST), and scores by PANSS, HAMD, MMSE, and CGI-S were evaluated twice: before and after the 28-th day of treatment. Activity of COX, GDH, GR, and GST was measured once in 23 women of 44-81 years old comprising the control group.

**Results:**

As compared with controls, only GDH activity was found significantly decreased (before and after treatment, p<0.001). Clusterisation of patients by enzymatic activities resulted in 3 clusters significantly different by COX, GDH and GST. Significant correlations were found between enzymatic activities and scores by psychometric scales: in the cluster 1 (n=9) baseline COX activity correlated with scores by PANSS positive subscale (R=0.9, p=0.001) and with scores by MMSE (R=-0.9, p=0.002); in the cluster 2 (n=12) GR activity after treatment negatively correlated with scores by PANSS (R=-0.9, p=0.001), PANSS negative subscale (R=-0.8, p=0.004), and CGI-S (R=-0.9, p=0.001).

**Conclusions:**

The revealed correlations between enzymatic activities and clinical parameters give hope on detection of useful biochemical markers which, after enlargement of patients’ group with late onset psychotic disorders, would be validated for prediction of the pharmacotherapy efficiency and outcome of treatment.

**Disclosure:**

No significant relationships.

